# Suture midface suspension

**DOI:** 10.1186/1746-160X-2-35

**Published:** 2006-11-01

**Authors:** Suat H Ugurbas, Robert A Goldberg, John D McCann, Norman Shorr, Rachna Murthy, Guy J Ben Simon

**Affiliations:** 1 Orbital and Ophthalmic Plastic Surgery Division, Jules Stein Eye Institute and Department of Ophthalmology, David Geffen School of Medicine, UCLA, Los Angeles, California, USA

## Abstract

**Objective:**

To describe a simple and effective facelift technique useful as an adjunct to other oculoplastic procedures

**Methods:**

Retrospective, non-comparative case series. Thirty five patients undergoing suture midface suspension from 1998 to 2000. Suspension sutures were passed from the nasolabial fold to the temporalis fascia to elevate the midface and the corner of the mouth.

**Results:**

A satisfactory and stable outcome is obtained in 2 years of follow up.

**Conclusion:**

Suture midface suspension is a safe and effective technique for the management of midface descent.

## Background

As our concept of facial rejuvenation has evolved, the midface has become an area of interest to oculoplastic surgeons. The midface is involved in the extended eyelid complex and also is affected by descent of the facial tissues during the aging process of the body. Drooping of facial skin and deepening of the nasolabial sulcus are characteristic features of midface descent. Several surgical methods that achieve vertical elevation are available to address this problem[1]. Today, the surgical techniques are shaped by an improvement in inert suture materials and interest for less invasive surgeries by the public. Herein, we describe and report the results of a simple and effective treatment for midface descent which is less invasive than the traditional deeper plane facelift surgeries. The purpose of the current paper is to describe two years follow up of 35 patients with mid-face descent of various causes who were operated using suture mid-face cable suspension at the division of orbito-facial surgery, Jules Stein Eye Institute.

## Methods

We reviewed the charts of 35 patients who underwent suture facelift surgery and at least 2 years of follow up after the operation. In this series, the procedure was performed either as primary surgery or as an adjunct to other oculoplastic procedures such as upper and lower blepharoplasty, endoscopic brow lift and neck liposuction.

All patients were assessed using pre- and post-operative full face photographs. Digital images were taken and recorded in the electronic medical record of the oculoplastic registry at the Jules Stein Eye Institute at each postoperative visit. Images were reviewed by two independent observers.

### Surgical technique

**Figure 1:**
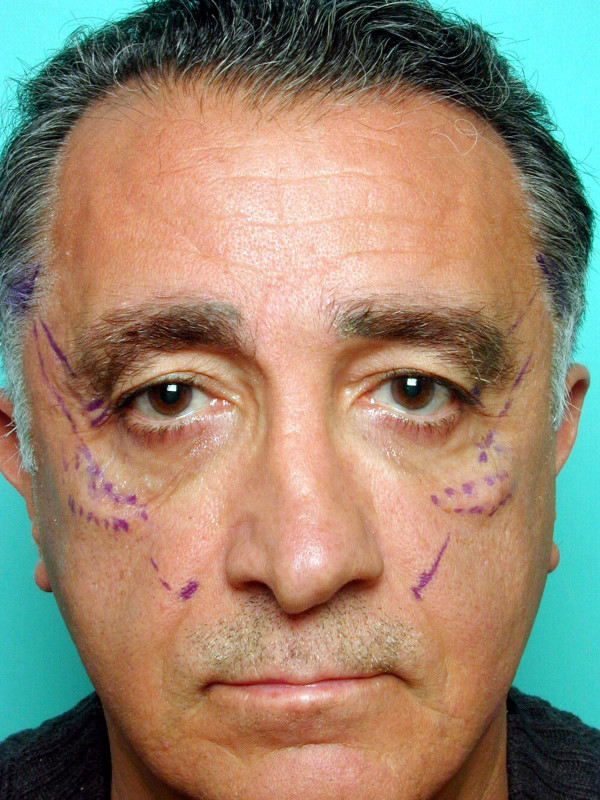
Midface suspension suture lines are marked.

The surgical procedure is carried out under local anesthesia with intravenous sedation. The midface suture suspension procedure is performed through a temporal incision. A marking is made 1 cm lateral to the nasal flare of the nostril on the nasolabial fold and a further marking 1 cm inferior to the previous, following the contour of the nasolabial fold. Two lines are drawn from the nasolabial fold markings to the temporal hair line. The first line passes 1 cm lateral to the lateral canthus and the second line runs parallel to the first, passing 1 cm lateral to it. These lines are extended to the temporal incision site which is marked 1 cm above the hairline (Figure 1).

**Figure 2:**
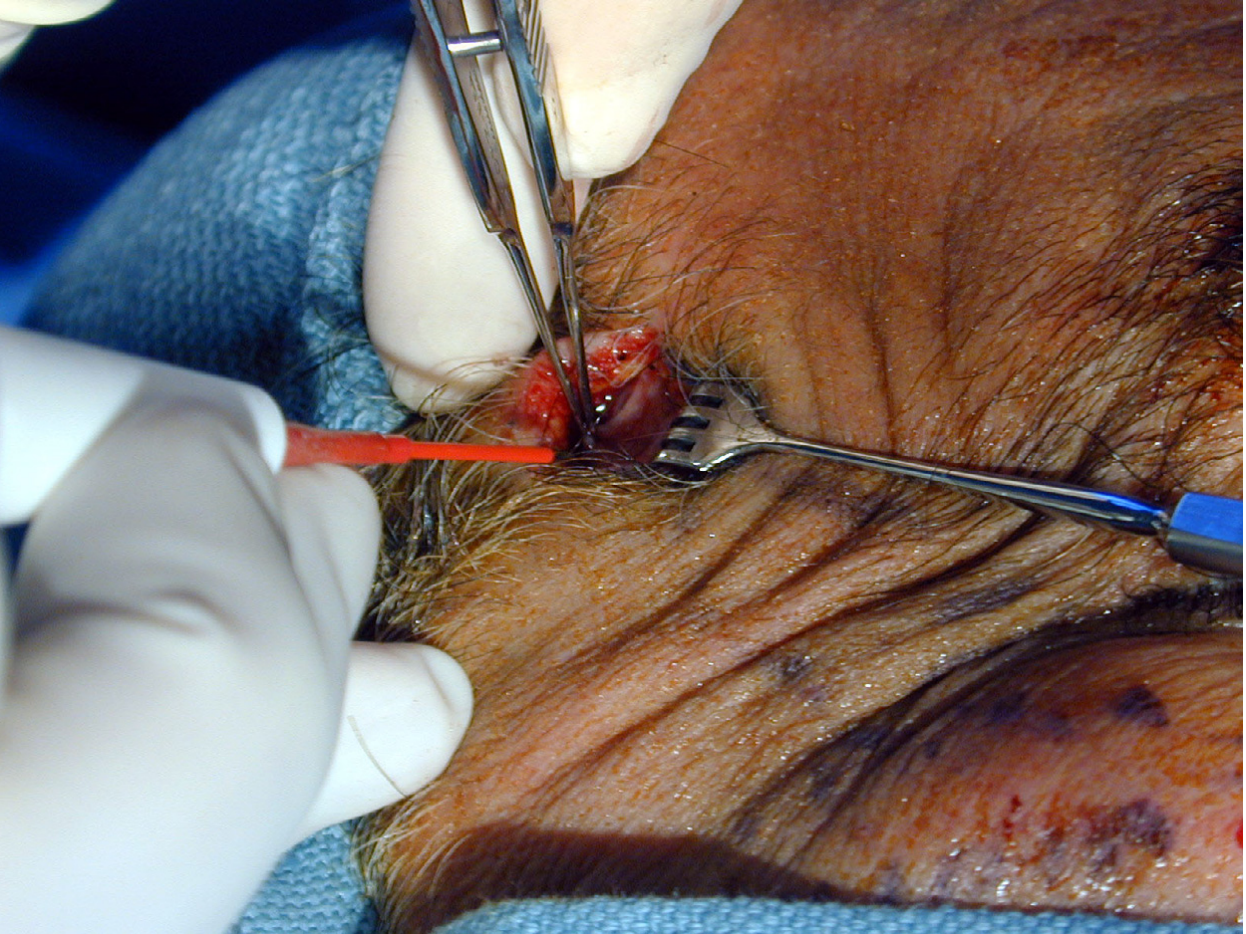
The incision is extended to the level of the deep temporalis fascia.

The temporal incision is made with a number 15 Bard Parker blade approximately 1 cm above the hairline area. Dissection is made directly through to the level of deep temporalis fascia (Figure 2). Two small stab wounds are made at the point of the nasolabial markings.

**Figure 3:**
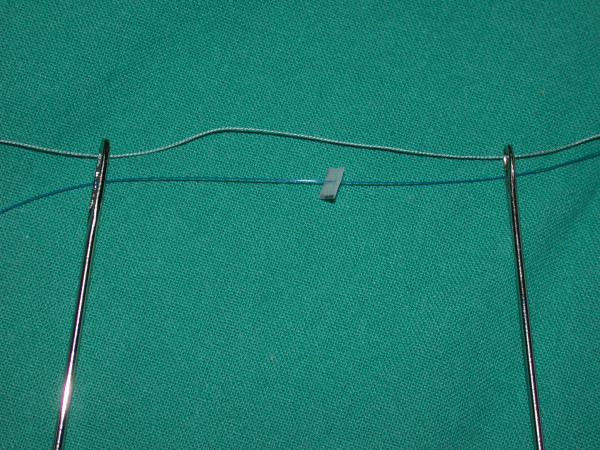
A hang-back suture is prepared with a Gore-Tex piece on the prolene suture.

**Figure 4:**
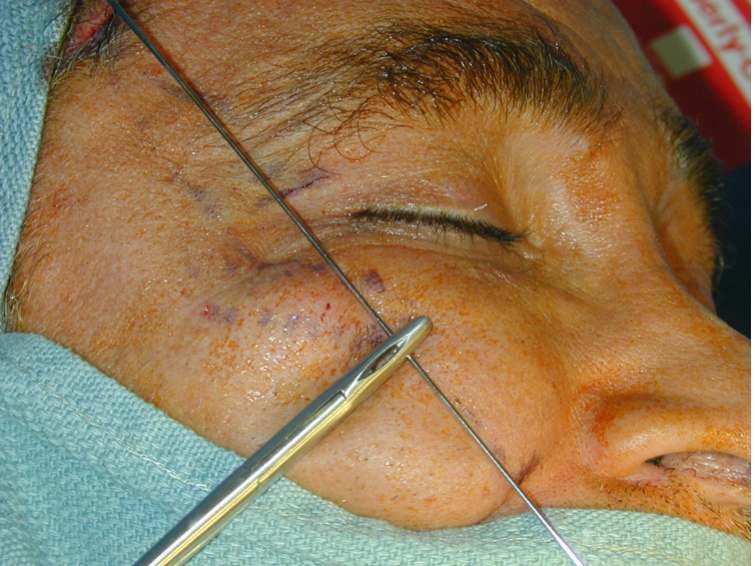
The Keith needle is shown on the marked line.

**Figure 5:**
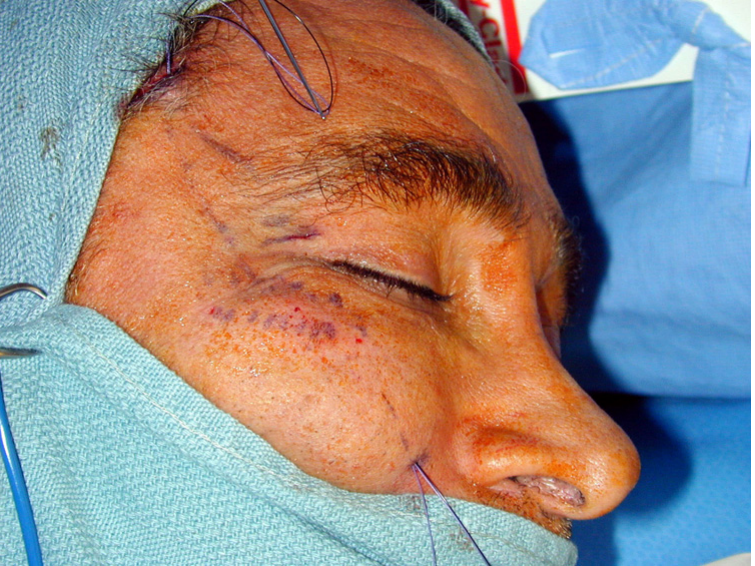
The Hang-back suture is passed through the SMAS plane.

Two hang-back sutures are prepared for each side of the face. A piece of Gore-Tex is prepared to the size of 4 × 4 mm and a 4-0 prolene suture passed through this Gore-Tex piece. A 4-0 prolene and 3-0 vicryl suture are threaded through the eye of two Keith needles (Figure 3). Each of the Keith needles are passed directly through the superior stab wound and advanced on the sub-SMAS (superficial musculoaponeurotic system) plane (Figures 4, 5). After the maxillary prominence, the first Keith needle is directed slightly superiorly and passed along the first marked line 1 cm lateral to the lateral canthus, coming out in the temporal incision site. The second needle is passed along the second marked line. After both Keith needles are retrieved from the temporal incision, the vicryl suture is gently pulled back and forth until it is externalised from the temporal incision, assuring there is no dimpling in the cheek. The 4-0 prolene suture is tied to the temporalis fascia adjusting the level of malar fat pad elevation. The second hang-back suture is passed in the same fashion starting from the lower stab incision in the nasolabial fold and the same procedure is repeated on the other side of the face. The temporal incision site is closed with a 5-0 chromic cat gut suture in a continuous fashion and the nasolabial stab wounds are closed with a single 6-0 cat gut suture.

Patients are given one gram of Cefazolin and the wound areas are dressed with an antibiotic ointment (Tobradex^® ^– tobramycin 0.3% and dexamethasone 0.1%). All surgeries were performed on outpatients' basis and under topical anesthesia (combination of lidocaine and bupivacaine).

## Results

**Figure 6:**
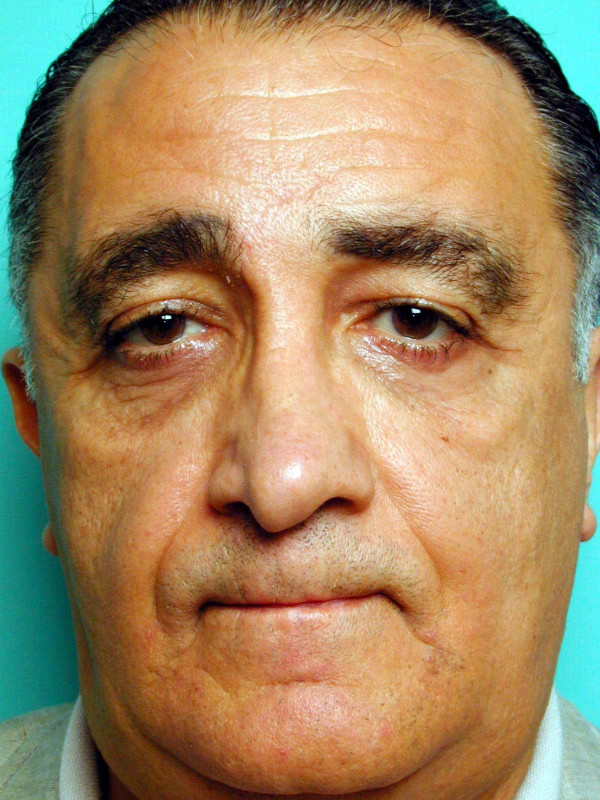
Pre-operative clinical photograph of the patient with midface descent.

**Figure 7:**
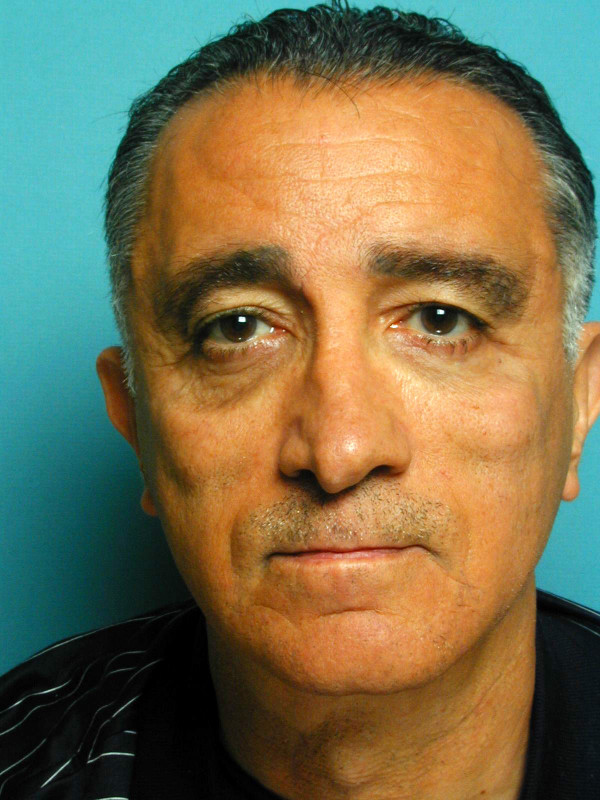
Postoperative clinical photograph of the patient (figure 6) after suture midface suspension.

Facial symmetry and a satisfactory midface lift were obtained in all patients with this procedure (Figures 6, 7). Local soft tissue reaction in the stab wound sites were observed in 2 patients immediately after the operation probably due to the Gore- tex. This reaction subsided quickly with the use of a tapered dose of oral steroids (Methylprednisolone dose package).

## Discussion

Facelift surgery is a part of facial rejuvenation. The main goal of surgery for facelift is to achieve a vertical elevation. It is in constant evolution, but is somewhat limited since the aging changes in the lower face are not completely addressed by current surgical techniques[2,3].

A combination of gravity and loss of elasticity and tone causes facial aging[4]. The sagging of the malar fat pad over the nasolabial folds contributes to a deeper appearance of these folds with time. Especially in patients at around the 40 year-age group, other signs of facial aging are not yet prominent. As classic techniques of facelift result in only modest improvement of deep nasolabial folds, in these cases a less invasive technique directly addressing the problem would be the procedure of choice. Suture midface suspension is especially helpful for the patient who is primarily concerned with midface descent.

Facelift surgery has some potential complications. Probably the most important one is damage to the facial nerve causing partial or complete facial palsy. Parotid duct injury may also occur. Flap necrosis and compromised wound healing causing scar tissue on the face are also important complications especially for smokers and vegetarian patients. Using an inert suture material to suspend the malar tissues above the nasolabial sulcus solves the problem in a simple and less complicated way. Softening of the nasolabial fold and lifting the malar fat pad can be achieved by these sutures.

On the other hand, suture midface suspension does not address all components of facial aging, such as fat atrophy. There is also a question of how long it lasts. The limitations of this technique should be explained to the patient before surgery.

A similar technique was previously described by Keller and associates[5] who evaluated 118 patients undergoing percuatneous malar fat pad elevation; at 3 months, all but two patients had a significant elevation of the malar fat pad of 3–7 mm. This procedure was associated with very little morbidity.

Suture midface suspension has the following advantages over deep plane facelifts: minimally invasive technique, performed under local anesthesia and relatively short procedure. However this technique has the following potential disadvantages: since there is no periosteal release and undermining the potential for significant and lasting elevation and repositioning of the malar mound may be limited; it is a blind procedure; hence it may carry the risk for neurovascular damage and finally stab incisions are done in visible, prominent areas. In addition skin irregularities (dimpling and bouncing) may appear at fixation points and sutures passed under the thin skin of the lateral canthal area may be palpable by the patients. Fixation to deep fascial plains such as deep temporalis fascia and passing the sutures at the SMAS levels may prevent these possible complications.

Our follow-up showed a reasonable cheek elevation and patient satisfaction. The results were more dramatic with moderate nasolabial folds and less dramatic in older patients with heavy nasolabial folds. This is as a result of inadequate suspension of the fat pad superiorly. We found this technique to be a useful adjunctive procedure in young patients who were undergoing surgery for facial rejuvenation and also in older patients who had previously undergone facelift surgeries. Further studies are warranties to evaluate the long term effect of suture midface suspension.
